# In a Circular Issued by the Board of Health

**Published:** 1885-10

**Authors:** 


### In a Circular Issued by the Board of Health

Of Bridgeport, Conn., the following directions about diet and cleanliness in cholera seasons are given "As regards special precautions against cholera, great care should be used in the matter of food and drink. Wholesome meats and vegetables, and ripe fruits, may be used freely; in fact the free use of acids in fruit or otherwise is supposed to act as a preventive. But under no circumstances should any decayed or stale vegetables, unripe fruit or tainted meat be allowed to be sold or used. The liability of water in the wells to become polluted by the contents of sewers and vaults, even if placed at a distance from them, should forbid the use of well-water for drinking purposes in all thickly settled places. Indeed, during a time of threatened cholera no water should be drank until it has first been boiled to destroy germs. It may afterwards be cooled by placing in contact with ice, but not by the addition of ice. Milk should be boiled before using for the same reason. The use of cathartic medicines should be entirely avoided except under the advice of a physician."—*Sanitary Engineer.*

